# A Review of the Use of Topical Calendula in the Prevention and Treatment of Radiotherapy-Induced Skin Reactions

**DOI:** 10.3390/antiox4020293

**Published:** 2015-04-23

**Authors:** Joyson Kodiyan, Kyle T. Amber

**Affiliations:** 1 Miller School of Medicine, University of Miami, 1600 Northwest 10th Avenue, Miami, FL 33136, USA; E-Mail: JKodiyan@med.miami.edu; 2 Department of Medical Education, Macneal Hospital, 3231 South Euclid Avenue, Suite 203 Berwyn, IL 60402, USA

**Keywords:** radiation induced skin toxicity, calendula, complementary and alternative medicine, radiodermatitis, radiation induced dermatitis, cancer

## Abstract

Calendula is a topical agent derived from a plant of the marigold family *Calendula Officinalis*. Containing numerous polyphenolic antioxidants, calendula has been studied in both the laboratory and clinical setting for the use in treating and preventing radiation induced skin toxicity. Despite strong evidence in the laboratory supporting calendula’s mechanism of action in preventing radiation induced skin toxicity, clinical studies have demonstrated mixed results. In light of the controversy surrounding the efficacy of calendula in treating and preventing radiodermatitis, the topic warrants further discussion.

## 1. Introduction

Upon receiving radiation therapy, up to 95% of patients suffer from radiation-induced skin damage, which can be significant enough to cause dose constraints [1]. In particular, acute radiodermatitis presents within 90 days of dosage administration with a blanchable, generalized erythema [2]. The National Cancer Institute ranks the severity of this dermatitis using a scale from 1 to 4 [3]. Acute skin toxicity and moist desquamation tends to occur in sites of dermal skin contact, such as the axilla or skin folds [4]. Management of this significant side effect is essential given its high incidence and considerably negative impact on quality of life [5]. Many patients search for complementary and alternative medicine (CAM) therapies to provide a solution for this condition [6]. In one particular study, a New Zealand regional cancer center found that 49% of a 200 patient sample receiving radiation therapy were using CAM [7]. At a radiation oncology clinic in Queensland, 38% of 101 patients were found to use CAM [6].

Soft silicon dressings have shown promise as effective barriers. Diggelmann *et al*. conducted a controlled clinical trial with Mepilex Lite dressings, showing significant improvement in skin reaction severity (up to 40%) over aqueous cream according to the Radiation-Induced Skin Reaction Assessment Scale (RISRAS) [8]. Mepilex Lite also had poor adherence to the axilla which is one of the most frequent areas for moist desquamation due to friction [4,9]. Similar beneficial findings were also found in another randomized controlled trial with Mepitel Film, where the film prevented moist desquamation significantly more than aqueous cream [10]. Zhong *et al*. demonstrated that application of Mepilex lite to moist desquamation significantly improved healing time and sleep disturbance compared salt water washings in patients with head and neck cancer receiving radiation therapy. Protective barriers including Safetac-based soft silicone dressings and Cavilon barrier films have also gained attention in the prophylaxis against radiation skin toxicity and moist desquamation [11].

Alcohol-free barrier films are thought to protect from moist desquamation due to radiation skin toxicity by reducing the rate of normal wear and tear from abrasion of superficial epidermal cells. Thus, the stem cells have sufficient time to repopulate the epidermis and prevent moist desquamation. Yet Graham *et al*. conducted a paired, double blind study on 318 patients receiving post-mastectomy radiotherapy comparing a moisturizing durable barrier cream to 10% glycerin, finding no statistically significant difference in grade 3 or greater skin toxicity between the two treatment arms. However, they attributed the absence of significant difference to the moisturizing durable barrier having a different acrylate terpolymer from a prior unblinded study that showed alcohol-free barrier film to have reduced moist desquamation compared to glycerine [12,13]. Several other treatment and preventive therapies for radiation-induced skin damage have been explored including washing [14], aloe vera [15], topical sucralfate [16], and corticosteroids [17]. Yet, the evidence has been conflicting on many of these modalities. Campbell *et al*. demonstrated a significant reduction in erythema when patients washed their skin either with or without soap [18]. Others trials by Roy *et al*. [19], Westbury *et al*. [20] , and Olsen *et al.* [21], however, found no significant difference between the two study arms in erythematous response. However, there was a notable difference in moist desquamation between the washing and non-washing groups in the study by Roy *et al*., 33% to 14% respectively.

Aloe vera, while containing numerous potent anti-inflammatory polyphenols [2] has failed to show any benefit when compared to inert gel or no treatment [22]. It has even been demonstrated to produce statistically significant worse outcomes with regards to dry desquamation and pain when compared to aqueous cream for irradiated breast cancer patients [23]. Topical sucralfate has likewise demonstrated mixed results as Wells *et al*. demonstrated greater erythema in patients using topical sucralfate compared to no cream [16]. Yet, Maiche *et al*. demonstrated that topical sucralfate had a significant improvement on erythema [24]. Elliot *et al*. conducted a trial of trolamine, a viscous organic compound, and found that it was not beneficial to best supportive care with regards to grade II or greater skin toxicity [25].

Topical steroids have demonstrated some efficacy in the prevention of radiodermatitis due to their anti-inflammatory qualities, preventing vasodilation and capillary permeability, thus inhibiting leukocyte migration [26]. In a randomized trial of patients receiving prophylactic mometasone *versus* emollient, those receiving mometasone had significantly less acute radiation dermatitis compared to those using emollient [26]. Schmuth *et al*. found many benefits with corticosteroid use compared to dexpanthenol, observing that patients treated with 0.1% methyprednisone had delayed and reduced both peak severity of radiation induced dermatitis and transepidermal water loss [27]. However, Potera *et al*. found no significant difference in acute and late skin toxicity between topical steroid prophylaxis and placebo [28].

Kumar *et al*. conducted a meta-analysis of topical therapies in the management of radiodermatitis, concluding that data was quite limited for most therapies and that radiation skin toxicity in multiple-site cancer patients did not favor treatment. However, Calendula, a topical agent derived from a plant of the marigold family *Calendula Officinalis*, did have limited evidence of effectively reducing grade 2 or greater skin toxicity in patients receiving breast irradiation [29]. On the other hand, Wong *et al.* noted the evidence to be weak for calendula’s effectiveness in treating radiation-induced skin toxicity [30], particularly noting the significantly lower adherence to calendula over trolamine [31]. In light of the controversy surrounding the efficacy of calendula in treating and preventing radiodermatitis, particularly grade II and greater erythema prevention, including moist desquamation, the topic demands further discussion. We performed a search of the Medline/Pubmed data base for calendula and radiation, radiotherapy and radiodermatitis.

## 2. Mechanism

Radiation exposure causes DNA damage from direct ionization of water molecules into radical oxygen species (ROS), which promote dimer formation, base alterations, and double strand breaks which can take up to 8 h to repair [32]. With fractionated radiation regimens, the stratum basale sustains damage, thus recruiting innate immune cells, particularly neutrophils and macrophages, which flood the stratum basale with ROS. Acutely, interleukin-1 (IL-1) and tumor necrosis factor-alpha (TNF-α) drive erythema and dermatitis, upregulating metalloproteases which degrade the dermis, particularly the basal layer [33,34,35]. Skin radiation exposure can further drive acute inflammation through upregulation of cell adhesion proteins such as intercellular adhesion molecule 1 (ICAM-1), thus promoting leukocyte adhesion and extravasation [36]. With further treatments, inflammation and ROS exposure is maintained through the adaptive immune response sustained by the T lymphocytes, ultimately leading to a deficit in the stratum basale and culminating in TGF-β which induces fibroblasts to cause chronic fibroblastic skin changes, dermatitis and both dry and wet desquamation [1,34,37,38,39,40].

Breast cancer patients receiving radiation therapy provide many insightful clues into the etiology of radiation skin toxicity. Severity of radiation induced skin toxicity can also be attributed to the type of radiation therapy, in particular with post-mastectomy radiation, where the target of therapy includes the skin. Severity can also vary with patient characteristics. In a prospective study of 110 patients receiving post mastectomy radiation therapy, Wright *et al*. found a statistically greater incidence of grade 4 or 5 skin toxicity in black patients *versus* non-black patients, in those greater than 50, in those with a BMI ≥ 25, and in postmenopausal patients. The authors of the study largely attributed the correlation between obesity and more severe skin toxicity to the difficulty to achieve dose homogeneity and the ability for skin folds, which are more prevalent with obesity, to behave as self-boluses of radiation to the skin [41]. Also, the association of increased skin toxicity with radiation therapy in African Americans is due to the higher rates of obesity in the community [41,42].

Pignol *et al*. conducted a double-blind study and found breast cancer patients treated with intensity-modulated radiation therapy (IMRT) experienced significantly less moist desquamation compared to standard treatment [43]. This difference can largely be attributed to the more homogenous radiation distribution in IMRT compared to standard radiation therapy with wedges which have a greater number of hotspots within the breast (radiation >10% of the prescribed dose), thus explaining the greater risk for acute dermatotoxicity [4,43]. Duration of treatment also has a significant impact on skin toxicity. Hypofractionated whole breast radiotherapy, which offers treatment over 3 weeks rather than the conventional 6 weeks [44], has statistically significant improvement in moist desquamation compared to conventional radiation therapy in 92 obese breast cancer patients, a population already at increased risk for radiation induced skin toxicity [45].

Calendula is able to prevent oxidative stress, making it theoretically an ideal treatment for radiodermatitis. This is thought to be through the numerous polyphenols contained in its extract. Polyphenols have many potentially therapeutic roles as antioxidants on the skin [46]. Braga *et al*. studied the pylene glycol extract of Calendula, finding that it interferes with neutrophil radical oxygen species (ROS) and radical nitrogen species (RNS) generation, particularly nitric oxide [47], at concentrations as low as 0.2 μg/mL, which was further confirmed by electron paramagnetic resonance (EPR) spectroscopy [48]. Nitric oxide, produced by neutrophil inducible nitric oxide synthase, can oxidize superoxide anions and form peroxynitrite anions which are highly cytotoxic due to oxidization of sulfhydryl groups on a multitude of proteins within cell nuclei [49]. Because neutrophils play a significant role in the free radical generation cascade, targeting this pathway can be of potential benefit. Alnuqaydan *et al*. demonstrated incubation of human non-cancer keratinocyte cell lines (HaCaT) with calendula extracts for 24 and 48 h increased survival of the human skin cell populations when exposed to hydrogen peroxide, an oxidative stressor, measured by DPPH assay and Folin-Ciocalteu assay. Additionally, doses of calendula less than or equal to 1% had no toxicity to HaCaT cells *in vitro* [50].

Hu *et al*. evaluated the impact of calendula on markers of inflammation found in the radiation-exposed skin of SKH-hr1 hairless mice. Calendula significantly reduced monocyte chemotactic protein-1, keratinocyte-derived chemokine, granulocyte colony-stimulating factor, IL-1 alpha, and vascular endothelial growth factor (VEGF), a set of cell signals elevated upon exposure to ionizing radiation. However, calendula was not found to have a significant impact on reducing erythema [51]. Preethi *et al*. found that calendula extract in male BALB/C mice led to reduced pro-inflammatory markers including TNF-α, IL-1β, (IL-6), interferon-gamma (IFN-γ), c-reactive protein (CRP), and cyclooxygenase-2 (COX-2). In parallel, the mice in the study models receiving the extract had significantly reduced paw edema [52].

Aside from antioxidant potential, Calendula also affects the skin architecture. By using a cutometer, Akhtar *et al*. found that calendula significant improved skin distensibility, direct markers of skin firmness, and viscoelasticity, which reflects water content in the epidermis and dermis [53,54].

## 3. Clinical Use

Given the potential promise of Calendula as an anti-inflammatory and antioxidant, there have been numerous developments in the clinical setting. Studies outside of radiodermatitis have shown some promise. Calendula has been noted to increase skin healing in oral mucositis second to 5-fluorouracil treatment compared to a control gel base [55]. Likewise, in a randomized study of 40 head and neck cancer patients receiving radiotherapy, those receiving calendula mouthwash had a significantly lower intensity of oropharyngeal mucositis compared to placebo at 2, 3 and 6 weeks. The efficacy of calendula in these instances has been attributed to flavonoids and phenolic substances, known for their radical scavenging and chelating abilities [56].

Pommier *et al*. conducted a large phase III randomized trial comparing prophylactic trolamine *versus* calendula in preventing acute dermatitis grade 2 or greater in breast cancer patients receiving radiotherapy. Their study demonstrated a significantly decreased incidence of acute dermatitis, interruptions in planned radiation schedule, and radiation-induced pain in patients using calendula compared to patients using trolamine. However, these results may have been influenced by the significantly lower adherence to the calendula treatment (30% of patients), possibly due to the cream’s consistency or difficulty in application [31]. As such, this study may in fact demonstrate the negative impact of trolamine on radiodermatitis rather than the efficacy of calendula. This study highlights the need for future studies to use vehicle controlled placebo in order to ensure the actual function of the active ingredient. In light of the limitations of Pommier’s study, the Multinational Association for Supportive Care in Skin Cancer (MASCC) Skin Toxicity Study Group does not currently recommend the use of calendula in radiation induced dermatitis [30].

In another randomized clinical trial of acute radiation-induced dermatitis, Sharp *et al*. compared Calendula cream with an aqueous cream in preventing dermatitis, evaluated by the Radiation Therapy Oncology Group/European Organization for Research and Treatment of Cancer (RTOG/EORTC) scale [57]. 411 breast cancer patients receiving adjuvant radiation therapy were randomized to either the calendula or the aqueous cream arm. Sharp found no significant difference between the two treatment arms in severe acute radiation skin reactions (ARSR), quality of life (HRQoL), adherence, and associated dermatologic symptoms such as pain, itching and burning. Thus Sharp *et al*. concluded that the skin product used had very little consequence on skin toxicity from radiation, attributing the lower rate of severe skin reactions (less than 25% by week 20 for both arms compared to 41% for the calendula arm and 63% for the trolamine arm in Pommier’s study [31]) to an improved toxicity profile of photon therapy and low number of smokers in the studied population. The use of an aqueous based topical solution is thus likely also superior to trolamine.

The adherence rates for the calendula cream are additionally of note, with an adherence rate of 86% and 84% for Pommier’s and Sharp’s trials, respectively [31,57]. In both studies, patients describe the Calendula cream to be more difficult in application and absorption when compared to the control arm. In light of these clinical trials for calendula, it is not surprising why its use continues to have mixed recommendations. In order to have more definitive evidence, vehicle controlled trials are essential. The lack of skin dosimetry verification for patients receiving radiation therapy is a key weakness in many of the studies comparing various treatments for skin toxicity, as having such information can allow for better risk stratification and greater likelihood of finding appropriate target populations for therapy. These smaller comparative studies also failed to account for skin type, quantity of skin fold, and area that was irradiated, all of which have a significant impact on dermatologic toxicity [41]. Due to limitations of sample size, many of the aforementioned studies failed to stratify patients according to obesity status and breast size, as these factors have been associated with worse dermatologic outcomes with radiation therapy in some studies [58], but not others [8,59,60,61]. Cumulative maximum dose to non-target sites was not verified in the studies of patients as well. Finally, it is difficult to compare various trials to eachother due to the inconsistent nature of reporting outcomes, particularly with regards to various skin toxicity scales used [62].

## 4. Safety

Overall, Calendula is clinically safe to use, though it does carry a small risk of inducing contact dermatitis, not unlike other topical polyphenols [63]. The Cosmetic Ingredient Review Expert Panel concluded the conventional concentrations of topical calendula formulations, including the flower extract ranging from concentrations of 0.0001% to 0.8% and flower oil ranging from concentrations of 0. 02% to 0.1%, are safe for use [64]. Various studies confirm that Calendula is not a sensitizer in the majority of patients and is benign in eczema patients [65,66,67,68].

## 5. Conclusions

In all, Calendula appears to be a safe topical therapy in the treatment and prevention of radiation induced skin-toxicity, however the evidence for its use remains weak. Its efficacy compared to other therapies is, however, still in question given the conflicting data reported in previous studies. The paucity of vehicle controlled trials in radiation induced skin toxicity severely limits interpretation of many topical therapies’ efficacy, as the moisturizing property of the vehicle may often play a larger role than the active ingredient.
